# Supplementary material for: Effect of d-cycloserine on fear extinction training in adults with social anxiety disorder
Source: PLoS One. 2019 Oct 17;14(10):e0223729. doi: 10.1371/journal.pone.0223729 (PMC6797442; doi:10.1371/journal.pone.0223729)
Supplement: S2 File — Sensitivity analyses were run with all available data to determine whether exclusion of non-conditioners affected the impact of DCS vs. PBO on extinction retention. Consistent with results when analyzing only conditioners, no main or interactive effects of group were seen during recall or renewal phases for SCR or US expectancy data. (HTML) [file pone.0223729.s002.html]

DCS SAD experiment all available data


# DCS SAD experiment all available data

# SCR

## Conditioning phase

```
#data
df.con <- df[df$phase == "CON" & df$stage == "late", c("subid", "group", "stimulus", "stage", "scr.sqrt")]
#summary
summary.con <- summarySE(df.con, measurevar = "scr.sqrt", groupvars=c("group", "stimulus", "stage"), na.rm = TRUE)
summary.con
```

```
#anova
results.con <- ezANOVA(data = df.con[complete.cases(df.con),], dv = scr.sqrt, within = .(stimulus), wid = subid, between = .(group), type = 3, detailed = TRUE, return_aov = TRUE)
anova_apa(results.con, effect = NULL, sph_corr = c("greenhouse-geisser"), es = c("pes"))
```

```
##           Effect                                              
## 1    (Intercept)  F(1, 67) = 84.81, p < .001, petasq = .56 ***
## 2          group  F(1, 67) =  0.00, p = .961, petasq < .01    
## 3       stimulus F(2, 134) = 19.20, p < .001, petasq = .22 ***
## 4 group:stimulus F(2, 134) =  0.80, p = .450, petasq = .01
```

## Extinction phase

```
#data
df.ext.all <- df[df$phase == "EXT", c("subid", "group", "stimulus", "stage", "scr.sqrt")]
#remove missing
ext.missing.id <- unique(df.ext.all[complete.cases(df.ext.all) == FALSE, "subid"])
df.ext <- df.ext.all[!(df.ext.all$subid %in% ext.missing.id),]
df.ext <- droplevels(df.ext)
#summary
summary.ext <- summarySE(df.ext, measurevar = "scr.sqrt", groupvars=c("group", "stimulus", "stage"), na.rm = TRUE)
summary.ext
```

```
#anova
results.ext <- ezANOVA(data = df.ext, dv = scr.sqrt, within = .(stimulus, stage), wid = subid, between = .(group), type = 3, detailed = TRUE)
anova_apa(results.ext, effect = NULL, sph_corr = c("greenhouse-geisser"), es = c("pes"))
```

```
##                 Effect                                             
## 1          (Intercept) F(1, 63) = 82.90, p < .001, petasq = .57 ***
## 2                group F(1, 63) =  0.49, p = .487, petasq < .01    
## 3             stimulus F(1, 63) =  4.24, p = .044, petasq = .06 *  
## 4                stage F(1, 63) = 20.42, p < .001, petasq = .24 ***
## 5       group:stimulus F(1, 63) =  0.37, p = .544, petasq < .01    
## 6          group:stage F(1, 63) =  0.61, p = .436, petasq < .01    
## 7       stimulus:stage F(1, 63) =  0.93, p = .338, petasq = .01    
## 8 group:stimulus:stage F(1, 63) =  1.90, p = .173, petasq = .03
```

## Recall phase

```
#data
df.rcl.all <- df[df$phase == "RCL",  c("subid", "group", "stimulus", "stage", "scr.sqrt")]
#remove missing
rcl.missing.id <- unique(df.rcl.all[complete.cases(df.rcl.all) == FALSE, "subid"])
df.rcl <- df.rcl.all[!(df.rcl.all$subid %in% rcl.missing.id),]
df.rcl <- droplevels(df.rcl)
#summary
summary.rcl <- summarySE(df.rcl, measurevar = "scr.sqrt", groupvars=c("group", "stimulus", "stage"), na.rm = TRUE)
summary.rcl
```

```
#anova
results.rcl <- ezANOVA(data = df.rcl, dv = scr.sqrt, within = .(stimulus, stage), wid = subid, between = .(group), type = 3, detailed = TRUE, return_aov = TRUE)
anova_apa(results.rcl, effect = NULL, sph_corr = c("greenhouse-geisser"), es = c("pes"))
```

```
##                 Effect                                                    
## 1          (Intercept)        F(1, 66) = 72.99, p < .001, petasq = .53 ***
## 2                group        F(1, 66) =  2.12, p = .150, petasq = .03    
## 3             stimulus  F(1.42, 93.63) =  6.28, p = .007, petasq = .09 ** 
## 4                stage        F(1, 66) = 33.64, p < .001, petasq = .34 ***
## 5       group:stimulus  F(1.42, 93.63) =  0.35, p = .630, petasq < .01    
## 6          group:stage        F(1, 66) =  0.75, p = .390, petasq = .01    
## 7       stimulus:stage F(1.63, 107.44) =  7.18, p = .002, petasq = .10 ** 
## 8 group:stimulus:stage F(1.63, 107.44) =  0.35, p = .662, petasq < .01
```

## Renewal phase

```
#data
df.rnl.all <- df[df$phase == "RNL",  c("subid", "group", "stimulus", "stage", "scr.sqrt")]
#remove missing
rnl.missing.id <- unique(df.rnl.all[complete.cases(df.rnl.all) == FALSE, "subid"])
df.rnl <- df.rnl.all[!(df.rnl.all$subid %in% rnl.missing.id),]
df.rnl <- droplevels(df.rnl)
#summary
summary.rnl <- summarySE(df.rnl, measurevar = "scr.sqrt", groupvars=c("group", "stimulus", "stage"), na.rm = TRUE)
summary.rnl
```

```
#anova
results.rnl <- ezANOVA(data = df.rnl, dv = scr.sqrt, within = .(stimulus, stage), wid = subid, between = .(group), type = 3, detailed = TRUE, return_aov = TRUE)
anova_apa(results.rnl, effect = NULL, sph_corr = c("greenhouse-geisser"), es = c("pes"))
```

```
##                 Effect                                                    
## 1          (Intercept)        F(1, 66) = 70.48, p < .001, petasq = .52 ***
## 2                group        F(1, 66) =  1.72, p = .194, petasq = .03    
## 3             stimulus       F(2, 132) =  6.54, p = .002, petasq = .09 ** 
## 4                stage        F(1, 66) = 26.33, p < .001, petasq = .29 ***
## 5       group:stimulus       F(2, 132) =  0.41, p = .667, petasq < .01    
## 6          group:stage        F(1, 66) =  0.04, p = .849, petasq < .01    
## 7       stimulus:stage F(1.82, 120.37) =  4.43, p = .016, petasq = .06 *  
## 8 group:stimulus:stage F(1.82, 120.37) =  1.55, p = .218, petasq = .02
```

# Expectancy

## Conditioning phase

```
#data
df.con.all <- df[df$phase == "CON" & df$stage == "late", c("subid", "group", "stimulus", "stage", "expectancy")]
#remove missing
con.missing.id <- unique(df.con.all[complete.cases(df.con.all) == FALSE, "subid"])
df.con <- df.con.all[!(df.con.all$subid %in% con.missing.id),]
df.con <- droplevels(df.con)
#summary
summary.con <- summarySE(df.con, measurevar = "expectancy", groupvars=c("group", "stimulus", "stage"), na.rm = TRUE)
summary.con
```

```
#anova
results.con <- ezANOVA(data = df.con, dv = expectancy, within = .(stimulus), wid = subid, between = .(group), type = 3, detailed = TRUE, return_aov = TRUE)
anova_apa(results.con, effect = NULL, sph_corr = c("greenhouse-geisser"), es = c("pes"))
```

```
##           Effect                                                
## 1    (Intercept)  F(1, 77) = 2019.96, p < .001, petasq = .96 ***
## 2          group  F(1, 77) =    0.64, p = .428, petasq < .01    
## 3       stimulus F(2, 154) =  123.27, p < .001, petasq = .62 ***
## 4 group:stimulus F(2, 154) =    0.85, p = .431, petasq = .01
```

## Extinction phase

```
#data
df.ext.all <- df[df$phase == "EXT", c("subid", "group", "stimulus", "stage", "expectancy")]
#remove missing
ext.missing.id <- unique(df.ext.all[complete.cases(df.ext.all) == FALSE, "subid"])
df.ext <- df.ext.all[!(df.ext.all$subid %in% ext.missing.id),]
df.ext <- droplevels(df.ext)
#summary
summary.ext <- summarySE(df.ext, measurevar = "expectancy", groupvars=c("group", "stimulus", "stage"), na.rm = TRUE)
summary.ext
```

```
#anova
results.ext <- ezANOVA(data = df.ext, dv = expectancy, within = .(stimulus, stage), wid = subid, between = .(group), type = 3, detailed = TRUE)
anova_apa(results.ext, effect = NULL, sph_corr = c("greenhouse-geisser"), es = c("pes"))
```

```
##                 Effect                                              
## 1          (Intercept) F(1, 75) = 660.25, p < .001, petasq = .90 ***
## 2                group F(1, 75) =   0.61, p = .436, petasq < .01    
## 3             stimulus F(1, 75) =  49.46, p < .001, petasq = .40 ***
## 4                stage F(1, 75) = 126.49, p < .001, petasq = .63 ***
## 5       group:stimulus F(1, 75) =   3.79, p = .055, petasq = .05 .  
## 6          group:stage F(1, 75) =   0.85, p = .359, petasq = .01    
## 7       stimulus:stage F(1, 75) =  42.35, p < .001, petasq = .36 ***
## 8 group:stimulus:stage F(1, 75) =   5.98, p = .017, petasq = .07 *
```

## Recall phase

```
#data
df.rcl.all <- df[df$phase == "RCL", c("subid", "group", "stimulus", "stage", "expectancy")]
#remove missing
rcl.missing.id <- unique(df.rcl.all[complete.cases(df.rcl.all) == FALSE, "subid"])
df.rcl <- df.rcl.all[!(df.rcl.all$subid %in% rcl.missing.id),]
df.rcl <- droplevels(df.rcl)
#summary
summary.rcl <- summarySE(df.rcl, measurevar = "expectancy", groupvars=c("group", "stimulus", "stage"), na.rm = TRUE)
summary.rcl
```

```
#anova
results.rcl <- ezANOVA(data = df.rcl, dv = expectancy, within = .(stimulus, stage), wid = subid, between = .(group), type = 3, detailed = TRUE, return_aov = TRUE)
anova_apa(results.rcl, effect = NULL, sph_corr = c("greenhouse-geisser"), es = c("pes"))
```

```
##                 Effect                                                
## 1          (Intercept)  F(1, 75) = 1373.97, p < .001, petasq = .95 ***
## 2                group  F(1, 75) =    1.41, p = .239, petasq = .02    
## 3             stimulus F(2, 150) =   29.09, p < .001, petasq = .28 ***
## 4                stage  F(1, 75) =  194.39, p < .001, petasq = .72 ***
## 5       group:stimulus F(2, 150) =    0.20, p = .818, petasq < .01    
## 6          group:stage  F(1, 75) =    0.32, p = .576, petasq < .01    
## 7       stimulus:stage F(2, 150) =   18.78, p < .001, petasq = .20 ***
## 8 group:stimulus:stage F(2, 150) =    0.97, p = .382, petasq = .01
```

## Renewal phase

```
#data
df.rnl.all <- df[df$phase == "RNL", c("subid", "group", "stimulus", "stage", "expectancy")]
#remove missing
rnl.missing.id <- unique(df.rnl.all[complete.cases(df.rnl.all) == FALSE, "subid"])
df.rnl <- df.rnl.all[!(df.rnl.all$subid %in% rnl.missing.id),]
df.rnl <- droplevels(df.rnl)
#summary
summary.rnl <- summarySE(df.rnl, measurevar = "expectancy", groupvars=c("group", "stimulus", "stage"), na.rm = TRUE)
summary.rnl
```

```
#anova
results.rnl <- ezANOVA(data = df.rnl, dv = expectancy, within = .(stimulus, stage), wid = subid, between = .(group), type = 3, detailed = TRUE, return_aov = TRUE)
anova_apa(results.rnl, effect = NULL, sph_corr = c("greenhouse-geisser"), es = c("pes"))
```

```
##                 Effect
## 1          (Intercept)
## 2                group
## 3             stimulus
## 4                stage
## 5       group:stimulus
## 6          group:stage
## 7       stimulus:stage
## 8 group:stimulus:stage
##                                                       
## 1        F(1, 74) = 941.80, p < .001, petasq = .93 ***
## 2        F(1, 74) =   0.00, p = .961, petasq < .01    
## 3 F(1.85, 136.72) =  22.70, p < .001, petasq = .23 ***
## 4        F(1, 74) = 135.15, p < .001, petasq = .65 ***
## 5 F(1.85, 136.72) =   0.17, p = .829, petasq < .01    
## 6        F(1, 74) =   0.07, p = .791, petasq < .01    
## 7       F(2, 148) =  16.84, p < .001, petasq = .19 ***
## 8       F(2, 148) =   1.08, p = .344, petasq = .01
```
