# Supplementary material for: Effect of d-cycloserine on fear extinction training in adults with social anxiety disorder
Source: PLoS One. 2019 Oct 17;14(10):e0223729. doi: 10.1371/journal.pone.0223729 (PMC6797442; doi:10.1371/journal.pone.0223729)
Supplement: Attachment — Submitted filename: DCS SAD Fear Conditioning Response to Reviewers1.docx [file pone.0223729.s004.docx]

Reviewer #1: The authors have addressed all my earlier comments. I have only one minor comment. The authors argue that the 62.5 % reinforcement rate "... was used ... because lower reinforcement rates create more uncertainty and lead to slower extinction [31,32], thus allowing more room for potential DCS augmentation effects." . As DCS is not believed to facilitate extinction learning, but rather to enhance extinction consolidation, I am not sure whether the second part of their argumentation is valid.

Response: We changed the sentence

“This reinforcement rate was used to replicate procedures from the previously validated paradigm used for this study [21,22], because lower reinforcement rates create more uncertainty and lead to slower extinction [31,32], thus allowing more room for potential DCS augmentation effects.”

To the following:

This reinforcement rate was used to replicate procedures from the previously validated paradigm used for this study [21,22], and to prevent the rapid extinction seen in protocols with 100% reinforcement [27,31].
